# Daily Reminders Increase Adherence to an Electronic Headache Diary (DMKG-App): Retrospective Longitudinal Cohort Study

**DOI:** 10.2196/73259

**Published:** 2026-09-14

**Authors:** Ruth Ruscheweyh, Lars Neeb, Bianca Raffaelli, Torsten Kraya, Tobias Freilinger, Thomas Dresler, Gudrun Gossrau, Tim Patrick Jürgens, Victoria Ruschil, Jörg Scheidt, Yannic Siebenhaar

**Affiliations:** 1 Department of Neurology, LMU University Hospital, LMU Munich, Marchioninistr. 15, Munich, 81377, Germany, 49 89-4400-52455; 2 Department of Neurology, Brandenburg Medical School, Theodor Fontane, University Hospital Brandenburg/Havel, Brandenburg/Havel, Germany; 3 Department of Neurology, Charité Universitätsmedizin Berlin, Corporate Member of Freie Universität Berlin, Humboldt-Universität zu Berlin, and Berlin Institute of Health, Berlin, Germany; 4 Clinician Scientist Program, Berlin Institute of Health, Berlin, Germany; 5 Department of Neurology, Hospital Sankt Georg Leipzig gGmbH, Leipzig, Germany; 6 Headache Center Halle, Department of Neurology, University Hospital Halle, Halle, Germany; 7 Department of Neurology, Klinikum Passau, Passau, Germany; 8 Department of Psychiatry and Psychotherapy, Tuebingen Center for Mental Health, University Hospital Tuebingen, Tuebingen, Germany; 9 German Center for Mental Health (DZPG), Partner Site Tuebingen, Tuebingen, Germany; 10 LEAD Graduate School & Research Network, University of Tuebingen, Tuebingen, Germany; 11 Interdisciplinary Pain Center, University Hospital and Faculty of Medicine Carl Gustav Carus, TU Dresden, Dresden, Germany; 12 Department of Neurology, Headache Center North-East, University Medical Center Rostock, Rostock, Germany; 13 Department of Neurology, KMG Klinikum Güstrow, Güstrow, Germany; 14 Center for rare diseases, University Hospital Tuebingen, Tuebingen, Germany; 15 Department of Neurology and Epileptology, Hertie Institute for Clinical Brain Research, University Hospital Tuebingen, Tuebingen, Germany; 16 Institute for Information Systems, University of Applied Sciences Hof, Hof, Germany

**Keywords:** headache, electronic headache diary, adherence, entry delay, attrition rate

## Abstract

**Background:**

The use of electronic headache diaries via smartphone apps has several advantages over paper and pencil diaries. Still, adherence is not always satisfactory. One potential strategy to improve adherence is the implementation of daily reminders, but their effect remains uncertain.

**Objective:**

The DMKG-App (the headache diary app of the German Migraine and Headache Society, DMKG) sends daily reminders to encourage user adherence. Due to a technical problem, reminders failed for a 14-day period in early 2023. This provided a unique opportunity to evaluate the effect of daily reminders on adherence to the headache diary in the form of a retrospective longitudinal observational cohort study.

**Methods:**

The failure period was compared to 14-day prefailure and postfailure periods and to corresponding periods of the previous year. We evaluated effects on the number of daily entries, delay to make the daily entry, and attrition rate, using ANOVA and logistic regression (α<.05). The effect of age, gender, headache frequency, and previous adherence was investigated using interaction analysis.

**Results:**

The DMKG-App had 4236 active users before reminders failed (females 81.6%, 95% CI 80.5‐82.8), age 39.8 (95% CI 39.4‐40.2) years. During the failure period, the number of daily entries fell by 11.0% (95% CI 9.2‐12.8). More specifically, entries per 14-day period significantly declined from 10.2 (95% CI 10.1‐10.3) prefailure to 9.1 (95% CI 9.0‐9.2) during the failure period, followed by a rise to 10.2 (95% CI 10.1‐10.3) during the postfailure period (*F*_2,11498_=79.2; *P*<.001). The delay to make the daily entry increased by 46.3% (95% CI 45.8‐46.8), from 1.90 (95% CI 1.89‐1.90) days prefailure to 2.78 (95% CI 2.77‐2.79) days during failure, and returned to 1.85 (95% CI 1.84‐1.86) days postfailure (*F*_2,39_=155.4*; P*<.001). In addition, there was a significant rise in attrition rate by 32.2% (95% CI 8.1‐61.7) (from 4.0%, 95% CI 3.4‐4.6, in the prefailure period to 5.2%, 95% CI 4.6‐5.9, in the failure period, returning to 3.9%, 95% CI 3.4‐4.5, in the postfailure period, *z*=3.10; *P*=.002). Interaction analysis revealed that the effect of reminder failure was larger in males than in females (regarding entry delay), in persons with lower compared to higher headache frequencies (regarding number of daily entries and entry delay), and in persons with higher vs lower previous adherence rates (regarding number of daily entries, entry delay, and attrition). There was no interaction between age and reminder failure.

**Conclusions:**

This is the first study investigating the effect of daily reminders in a digital headache diary app. Results suggest that reminders are an effective means to improve adherence. Males, persons with low headache frequencies, and those with high adherence during the presence of reminders seem to benefit most. These results are important additions to our knowledge on compliance with digital health apps.

## Introduction

Headache diaries are important tools for diagnosis and treatment evaluation in primary headaches [1]. Indeed, it has been shown that self-reported retrospective estimations of headache frequency are highly unreliable. In a study from the Leiden Headache Center, for example, the average difference between retrospectively self-reported and diary-assessed monthly migraine days was 4.7 (SD 5.0) days per month, with both overestimation and underestimation occurring [2]. Paper-and-pencil diaries have been used for a long time and have the advantage of being simple and free from technical requirements. On the other hand, they can get lost, may not be at hand when needed, and patients may sometimes fill in missing days shortly before the appointment, resulting in recall bias [3,4].

While some patients prefer paper diaries, smartphone-based electronic headache diaries have gained popularity in recent years. At a time when most people carry a smartphone, and with the majority of headache patients being relatively young and comfortable with digital technology, smartphone apps have several advantages [4]. The smartphone is always at hand, recall bias can be reduced by allowing retrospective entries only for a defined period, and automatic summaries give a quick overview to be shared with the physician [5,6]. Patients can be guided through data capture, and automated data validation improves data quality [6]. It has been shown that electronic diaries are superior to paper diaries in several respects (better validity, less missing data, and larger patient satisfaction [4,7]). Data can also more easily be used for large-scale research, as recently shown in an analysis of acute medication rating based on more than 270,000 users [8]. In the future, data from headache diary apps might be integrated with other health data, with possible applications, for example, for attack prediction [9].

Still, adherence to the diary is key to headache evaluation, and prompt entries increase accuracy by decreasing recall bias [2]. One attempt to increase adherence to headache apps is to send users daily reminders.

Different behavioral or motivational theory approaches offer explanations of how reminders may improve the target behavior (making the daily entry) [10]. For example, the COM-B model focuses on capability (C), opportunity (O), and motivation (M) as conditions of behavior (B) [11]. Regarding a diary app, the person needs to be proficient in handling a smartphone app (C), must have access to a smartphone and internet connection (O), and needs the motivation to fill in the diary. In this model, reminders might make the original motivation salient again, increasing the frequency of the behavior. The behavioral model developed by Fogg [12] introduces an additional element by assuming that the building blocks of behavior are the combination of motivation, ability, and triggers. A reminder is a prototypical example of a trigger, prompting existing motivation and ability into behavior. There also is the traditional concept of intrinsic vs extrinsic motivation, with the former describing that behavior is motivated “because it is inherently interesting or enjoyable,” and the latter behavior that is motivated “because it leads to a separable outcome” [13]. A well-designed reminder might make the daily entry enjoyable. On the other hand, it may help to achieve a goal (headache documentation), so reminders may act on both intrinsic and extrinsic motivation. The self-determination theory is a modern theory of intrinsic motivation [14] and emphasizes that specific psychological needs (ie, autonomy, competence, and relatedness) must be satisfied to motivate human behavior. Reminders may promote a feeling of autonomy, as participants can, but do not have to, act upon them. Reminders may increase the feeling of competence by making it easier to reach the goal. They could also be specifically designed to create a sense of social relatedness. Also, concepts from digital literacy research may be useful for understanding the function of reminders, as this research suggests that both the user’s ability and the system’s usability must be present for a digital tool to be used effectively. In this framework, reminders increase the usability of a headache diary, as they make the diary easier to use [15].

The electronic headache diary of the German Migraine and Headache Society (DMKG; the smartphone app is called “DMKG-App”) uses daily reminders [16]. A technical problem in early 2023 resulted in a 2-week period during which no reminders were sent. This allowed us to analyze if the absence of reminders had an effect on (1) the number of days recorded in the app (daily entries), (2) the delay to record the daily entry (entry delay), or (3) a disproportionately high number of users stopping use of the app altogether (attrition rate). In addition, we investigated whether age, gender, headache frequency, and previous adherence influence the reaction to reminder failure.

## Methods

### Study Design

This is a retrospective longitudinal cohort study using data from a smartphone headache diary app that sends daily reminders to make the daily entry. For technical reasons, reminders failed for an approximately 2-week period in early 2023. We investigated how adherence was affected by reminder failure by comparing the failure period with 2 periods with intact reminders and with the analogous periods from the previous year.

### Setting and Data Sources

The analysis used data from the smartphone headache diary app of the German Migraine and Headache Society (DMKG-App), available in Germany for Android and iOS since June 2020 for users aged at least 16 years. In the DMKG-App, users are asked to record every day either as “no headache” or “headache.” In the case of “headache,” additional headache characteristics are assessed, which were not analyzed here. A reminder to make the daily entry is sent as a smartphone push notification every day around 8 PM (fixed time), reading, “Don’t forget your headache diary. Your DMKG-App is ready for today’s entry.” Entries can be made retrospectively for a maximum of 14 days in the past as a compromise between reducing recall bias and maintaining app usability.

### Participants

The DMKG-App is available in Germany for users aged at least 16 years who use the app either for their personal headache documentation or to support their headache treatment. We included participants fulfilling the criteria of being an “active user” during one of the periods of interest (see “Statistical Analysis” section below).

### Ethical Considerations

The DMKG-App is part of the larger project “DMKG Headache Registry” [16] that has been approved by the ethics committee of the Ludwig-Maximilians-University Munich (Nr. 20‐004), complies with European and German Data Protection laws, and is registered with the German Clinical Trials Register (DRKS 00021081). The DMKG-App is freely available for use by persons with headaches in Germany.

Each participant provided informed consent for the use of their data for headache research. As health data constitute a special category of personal data, participants provided explicit consent to the processing and scientific use of their data in accordance with Article 9(2)(a) of the General Data Protection Regulation (GDPR) [17]. The present analysis also includes data from adolescents aged at least 16 years. Article 8(1) of the GDPR provides that, where an information society service is offered directly to a minor, individuals aged at least 16 years may provide consent themselves [18]. Although European Union (EU) Member States may establish a lower age threshold, Germany has not adopted such a lower threshold. The German Data Protection Conference also addresses these requirements in its Short Paper No. 20 [19] on consent under the GDPR, which refers both to the requirement of explicit consent for the processing of special categories of personal data and to the specific conditions under Article 8 of the GDPR for children’s consent in relation to information society services. The consent procedure for DMKG-App use was designed on this legal basis. Accordingly, participants aged 16 or 17 years provided the required informed and explicit consent themselves, and no additional consent from a parent or legal guardian was obtained.

Only anonymized data were used for the present analysis, and only aggregated results are reported. Individual users cannot be identified from any part of this manuscript.

### Analyzed Failure and Reference Periods

The failure to send reminders occurred following the release of a new app version (2.0.0) on December 17, 2022, and was corrected with version 2.0.4 released on February 10, 2023. However, after an entry in the app is made, reminders are preplanned for the next 6 (42 d) weeks and then work independently of the app version. This resulted in active app users experiencing failure of reminders only toward the end of January 2023. In addition, not all users updated to the new version immediately. Therefore, although reminders slowly started to fail beginning on December 17, a general failure occurred between January 28 and February 10, 2023. Therefore, we used this 14-day period for analysis. For comparison, we chose 14-day reference periods before December 17 (December 3-16, 2022) and in equal distance after the failure period (March 25 to April 07, 2023). We called these analysis periods the “prefailure,” “failure,” and “postfailure” period, respectively.

To exclude the possibility that circannual variations affected the results, we also analyzed the same time periods in the preceding year (ie, precontrol: December 3-16, 2021; control: January 28 to February 10, 2022; postcontrol: March 25 to April 07, 2022).

### Variables

Users can record every day either as “no headache” or “headache,” or make no entry. Both “no headache” or “headache” days counted as recorded days. From this measure, we calculated 3 outcome parameters for each period: the number of recorded days (“daily entries”), the delay to make the daily entry (measured in days; the app allows making entries with a maximum delay of 14 d), and the attrition rate (users who had been active before the period but made no further entry during or after that period).

In addition, we used age, gender, headache frequency, and previous adherence categories to analyze if these variables modulated the effect of reminder failure. For age, 2 groups were defined by median split (younger <37 y and older ≥37 y). For headache frequency, categories were chosen according to migraine frequency categories [20] (low, medium, or high frequency: 0‐7 d/mo; 8-14 d/mo; ≥15 d/mo, respectively) and determined from the entries in October 2022 (ie, before the prefailure period). Only participants who had made more than 70% of the possible entries in this time frame were included to reduce bias in headache frequency detection related to low adherence. For previous adherence, the number of entries in October 2022 was used. Most participants made daily entries (31 days, which we termed: “high previous adherence”), the remainder was more or less evenly distributed between 0‐14 entries (“low previous adherence”) and 15‐30 entries (“medium previous adherence”).

There were no missing data, as age and gender are mandatory entries in the DMKG-App, and the fact that some days were recorded and others not was the subject of our analysis.

### Statistical Analysis

Statistical analysis was made in R (R Foundation for Statistical Computing; version 4.4.0). 95% CIs are given. *P*<.05 was considered significant. Post hoc tests comparing the 3 periods were corrected for 3 comparisons with Bonferroni Holm correction, and corrected *P* values (*P*_c_) are reported [21].

As the number of app users changes continuously (new users joining and previous users ceasing to use the app; for the DMKG-App there currently is a net increase in users), we based our analysis on the number of active app users on a specific day, defined as users with at least 2 entries in the app, 1 before the analyzed day and 1 on or after the analyzed day.

**Figure 1.**
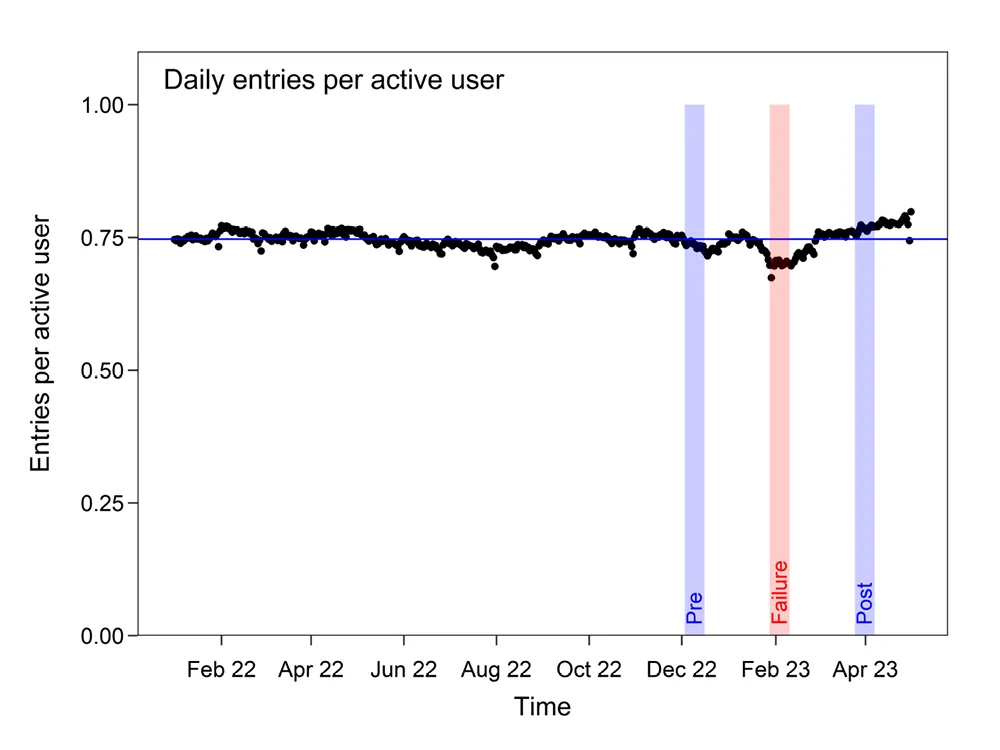
Time course of the number of daily entries with respect to active app users. This was a longitudinal analysis of data from the Deutsche Migräne- und Kopfschmerzgesellschaft e.V. (German Migraine and Headache Society, DMKG) app, a headache diary app freely available in Germany, comparing a period where no daily reminders were sent with periods with reminders. A significant dip in the daily entry rate is seen during the period where no reminders were sent (failure). Each data point represents 1 day. Please note that the red bar denotes the absolute failure period. For some users, failure occurred earlier and/or lasted longer (see “Methods” section), explaining why the reduction of daily entries extended beyond the failure period. Prefailure and postfailure periods are marked by blue bars.

To analyze the number of recorded days (“daily entries”) during the different periods, we compared the number of entries within the respective 14-day period per active user between periods (prefailure, failure, and postfailure) using ANOVA followed by a Wilcoxon rank sum test with continuity correction. For a day-by-day graphical illustration, the number of entries on every day was set into proportion to active users on this day (Figure 1).

For the delay in recording the daily entry (“entry delay”), the number of days between the recorded day and the day the entry was made was calculated, averaged within periods and compared between periods using ANOVA followed by Welch *t* tests (2-tailed). If the entry was made on the day itself, the delay was 0. Only days with an entry were considered.

To analyze the proportion of users stopping app use (“attrition rate”) during the different periods, all active users on the day before the start of the respective period were considered. For every day during the analyzed period, users that had not made any further entry on that day or any day after this (up to data closure on June 20, 2023) were considered to have stopped app use. Proportions of users having stopped app use at the end of the respective period were compared between periods using logistic regression.

To analyze if age, gender, headache frequency, and previous adherence modulated the effect of reminder failure on daily entries, delay, and attrition rate, categorical variables as defined above were included in the respective ANOVA or logistic regression.

## Results

The number of active users at the start of the reminder failure period was 4236. A total of 3460 (81.6%, 95% CI 80.5‐82.8) were female, and the average age was 39.8 (SD 12.8, 95% CI 39.4‐40.2) years.

### Number of Recorded Days in the Diary (Daily Entries)

Figure 1 shows the course of daily entries in relation to active users, displaying a remarkable dip during the period where no reminders were sent. Active users made 10.2 (SD 4.3, 95% CI 10.1‐10.3) of 14 possible entries during the prefailure period, 9.1 (SD 4.6, 95% CI 9.0‐9.2) during the failure period, and 10.2 (SD 4.3, 95% CI 10.1‐10.3) in the postfailure period. There was a significant difference between the 3 periods (*F*_2,11498_=79.2; *P*<.001). Post hoc tests revealed significant differences between the prefailure and failure periods (*z*=11.9; *P*_c_<.001) and between the failure and postfailure periods (*z*=12.5; *P*_c_<.001) but not between the prefailure and postfailure periods (*z*=0.1; *P*_c_=.90).

This was not an effect of time of the year, as no such effect was seen in the respective periods in the previous year (prefailure: mean 10.4 (SD 4.0, 95% CI 10.2‐10.6) entries; failure: mean 10.5 (SD 4.1, 95% CI 10.3‐10.7) entries; postfailure: mean 10.5 (SD 42, 95% CI 10.3‐10.7) entries; *F*_2,5134_<0.1; *P*=.91).

Thus, there was a reduction of daily entries per active user of 11.0% (95% CI 9.2‐12.8, from 10.2 to 9.1 entries per 14-d period) from the prefailure to the failure period.

### Delay to Make the Daily Entry

**Figure 2.**
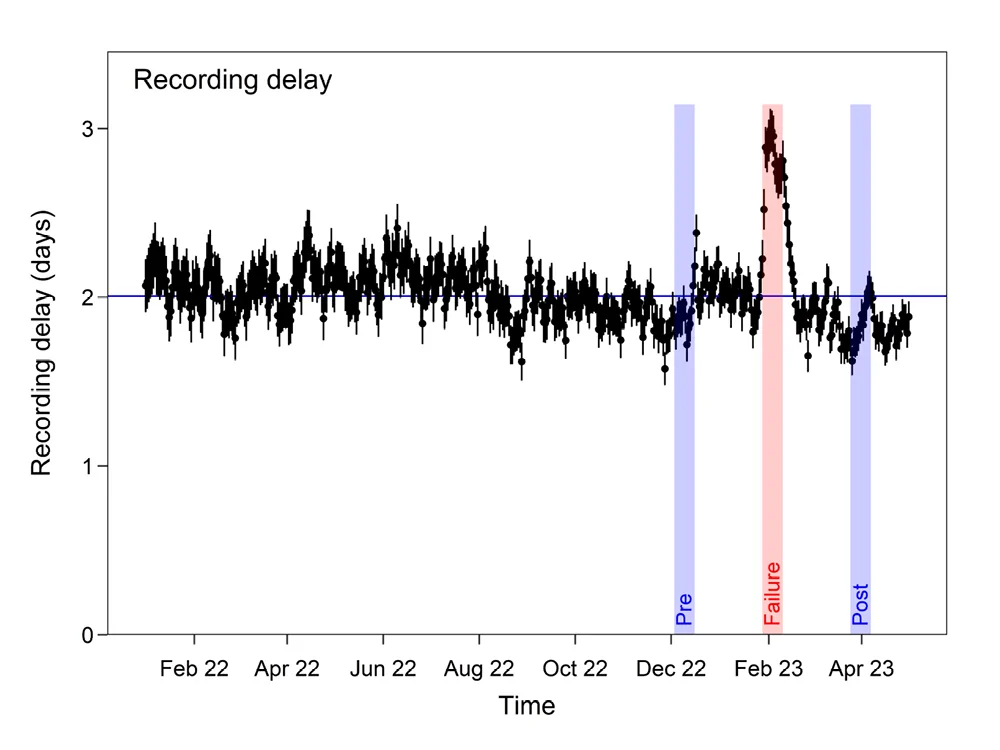
Time course of the delay in recording the daily entry. This was a longitudinal analysis of data from the Deutsche Migräne- und Kopfschmerzgesellschaft e.V. (German Migraine and Headache Society, DMKG) app, a headache diary app freely available in Germany, comparing a period where no daily reminders were sent with periods with reminders. A significant rise in delay is seen during the period where no reminders were sent (failure). Each data point represents 1 day; error bars correspond to SDs. Prefailure and postfailure periods are marked by blue bars.

Figure 2 illustrates that the typical average delay in recording was around 2 days, which sharply increased to almost 3 days during the failure period. Specifically, average delays were 1.90 (SD 0.12, 95% CI 1.89‐1.90) days in the prefailure period, 2.78 (SD 0.20, 95% CI 2.77‐2.79) in the failure period, and 1.85 (SD 0.14, 95% CI 1.84‐1.86) days in the postfailure period. This was highly significant between periods (*F*_2,39_=155.4; *P*<.001). Post hoc tests revealed significant differences for comparison of prefailure to failure (*t*_20.5_=−14.0; *P*_c_<.001) and failure to postfailure (*t*_22.7_=14.1; *P*_c_<.001) but not prefailure vs postfailure (*t*_25.3_=1.0; *P*_c_=.24).

Again, this was not due to circannual variation, as the values for the respective periods of the preceding year were: precontrol mean 2.08 (SD 0.07, 95% CI 2,08‐2,08), control mean 2.03 (SD 0.10, 95% CI 2.02‐2.04) and postcontrol mean 1.97 (SD 0.11, 95% CI 1.96‐1.98). While ANOVA detected significant differences between periods (*F*_2,39_=5.9; *P*=.006), the control period was not significantly different from either precontrol or postcontrol periods (precontrol vs control periods: *t*_23.5_=1.68; *P*_c_=.11; control vs postcontrol: *t*_25.8_=1.72; *P*_c_=.19). However, there was a small but significant reduction from precontrol to postcontrol (*t*_22.4_=3.5; *P*_c_=.002) that was not further explored.

The increase of recording delay from the prefailure period (1.90) to the failure period (2.78) corresponds to a relative increase of 46.3% (95% CI 45.8‐46.8).

### Users Stopping App Use (Attrition Rate)

**Figure 3.**
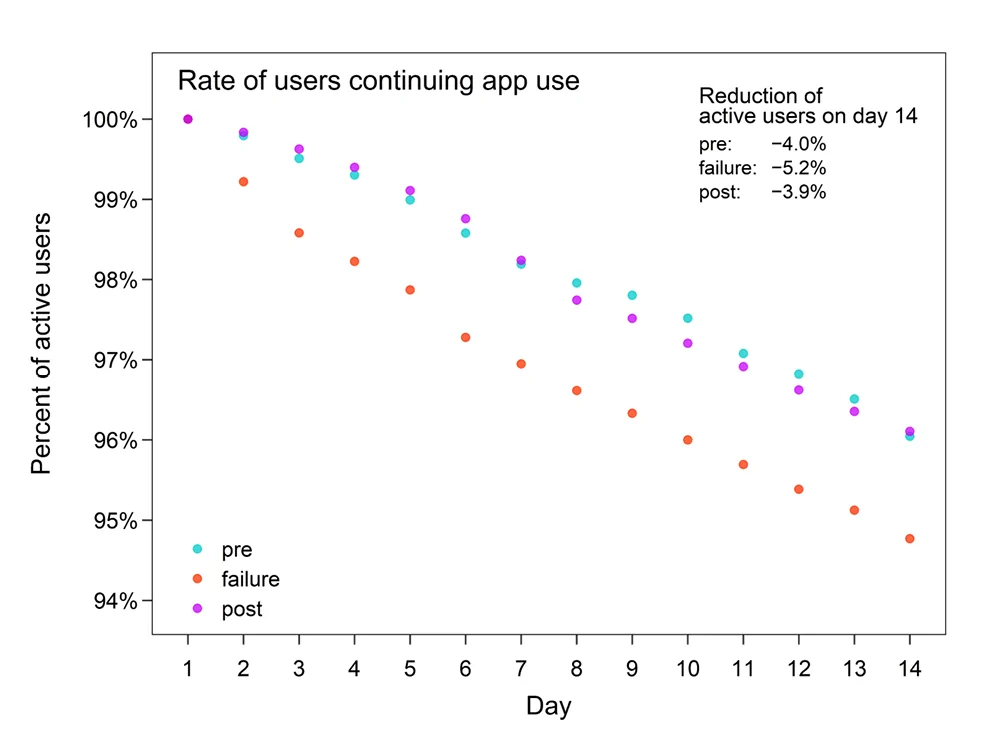
Attrition rates during failure of reminders vs before and after failure of reminders. This was a longitudinal analysis of data from the Deutsche Migräne- und Kopfschmerzgesellschaft e.V. (German Migraine and Headache Society, DMKG) app, a headache diary app freely available in Germany, comparing a period where no daily reminders were sent (failure) with 2 periods with intact reminders (prefailure and postfailure). The rate of previously active users who continued using the app during the failure period compared to the prefailure and postfailure periods.

There is always a certain number of users stopping app use. From all users classified as active before the day where reminders failed, a total of 5.2% (220/4236 users, 95% CI 4.6‐5.9) stopped using the app for good (ie, made no further entry after that). In comparison, in the prefailure and postfailure periods, 4.0% (155/3886, 95% CI 3.4‐4.6) and 3.9% (189/4845, 95% CI 3.4‐4.5) stopped app use (Figure 3). Logistic regression revealed significant differences between periods (prefailure vs failure: *z*=2.8; *P*=.006; postfailure vs failure: *z*=3.10; *P*=.002). Also, in the previous year, attrition rates were 2.8% (49/1735, 95% CI 2.1‐3.7), 3.0% (58/1929, 95% CI 2.3‐3.9) and 2.9% (64/2192, 95% CI 2.3‐3.7) during the precontrol, control, and postcontrol periods, without significant differences (prefailure vs failure: *z*=0.1; *P*=.91; postfailure vs failure: *z*<0.1; *P*=.99).

Thus, there was an excess loss of 1.2% (5.2% in the failure period vs 4.0% in the prefailure period) of active users within the period with notification failure, or, put differently, the loss of active users during the failure period increased by 32.2% (from 4.0% to 5.2%, 95% CI 8.1‐61.7).

### Interaction With Age

There was no significant interaction between age and period, neither for the number of recorded days nor for the delay to make the daily entry, nor for attrition rates (see Table S1 in Multimedia Appendix 1 for details).

### Interaction With Gender

The increase of the delay to make the daily entry from the prefailure to the failure period was significantly lower in females than in males (+0.85, 95% CI 0.84‐0.86) days vs +1.03 (95% CI 1.00‐1.06) days; interaction between gender and period: *F*_2,78_=5.0; *P*=.009). There was no interaction between gender and period regarding the number of recorded days or the attrition. Details are listed in Table S1 in Multimedia Appendix 1. In summary, females were less sensitive than males to reminder failure regarding the delay to make the daily entry.

### Interaction With Headache Frequency

Results are summarized in Table S2 in Multimedia Appendix 1. For the number of recorded days, there was an interaction between period and headache frequency (*F*_4,5536_=3.3; *P*=.01), with the prefailure to failure reduction being significantly larger in the low frequency group (−1.49, 95% CI −1.10 to −1.76 entries per 14 d) than in the 2 other groups (medium: −0.79, 95% CI −0.42 to −1.16; *P*=.004; high: −0.88, 95% CI −0.39 to −1.37; *P*=.04). For the delay to make the daily entry, there was an interaction between period and headache frequency (*F*_4,117_=18.9; *P*<.001), with the prefailure to failure increase being larger with lower headache frequencies (low: +1.17, 95% CI 1.15‐1.19; medium: +0.87, 95% CI 0.85‐0.89; high: +0.60, 95% CI 0.58‐0.62 days; all *P*<.001). For the attrition rate, there was no interaction between period (pre vs failure) and headache frequency (*z*=−0.64; *P*=.53).

In summary, patients with lower headache frequencies were more sensitive to reminder failure regarding the number of recorded days and the delay to make the daily entry.

### Interaction With Previous Adherence

Results are summarized in Table S2 in Multimedia Appendix 1. For the number of recorded days, there was an interaction between period and previous adherence (*F*_4,6947_=5.8; *P*<.001), with the prefailure to failure decrease being larger in the high compared to the low previous adherence group (−1.21, 95% CI −1.44 to −0.98 d vs +0.13, 95% CI −0.60 to 0.86; *P=*.002). For the delay, there was an interaction between period and previous adherence (*F*_4,117_=10.7; *P*<.001), with prefailure to failure increases of the delay being largest in the high previous adherence group (low: +0.77, 95% CI +0.73 to +0.81; medium: +0.81 (95% CI +0.78 to +0.84); high: +0.98 (95% CI +0.97 to +0.99) days; all 3 comparisons significant, Table S2 in Multimedia Appendix 1). For the attrition rate, there was a significant interaction between period (pre vs failure) and previous adherence (*z*=−4.22; *P*<.001). Excess attrition rates (pre vs fail) were highest in the high previous adherence group (low: −1.9% (95% CI −4.4 to +0.5); medium: −0.5% (95% CI −2.8 to +1.7); high: +2.7% (95% CI +1.6 to +3.8); both comparisons with high previous adherence were significant, Table S2 in Multimedia Appendix 1).

In summary, patients with high adherence during the presence of daily reminders were most sensitive to failure of these reminders.

## Discussion

### Principal Findings

This study demonstrates that daily reminders can significantly contribute to the adherence to electronic headache diaries. Absence of reminders during a 14-day period resulted in a measurable decline in daily entries by 11% and an increase of daily entry delays by 46% and attrition rates by 30%. Moreover, males, participants with lower headache frequencies, and with higher previous adherence showed a larger decrease in adherence in reaction to the reminder failure, while age groups did not differ in reaction to reminder failure.

The present results show that daily reminders indeed improved adherence to the DMKG-App electronic headache diary. From the different behavioral and motivational theories, Fogg’s model may be best suited to understand the effect of reminders. In accordance with this model, smartphone reminders serve as external triggers, sometimes also called signals or nudges, to perform the desired behavior and can be seen as elements of persuasive technology [12,22]. Behavioral model developed by Fogg additionally lists ability and motivation as prerequisites for behavior and the digital literacy literature emphasizes that both ability and usability must come together for a digital tool to be used effectively [15]. In the DMKG-App, reminders are implemented as push notifications taking the user directly to the daily entry. Failure of reminders would be supposed to reduce usability [15] by making the daily entry more difficult, as the user has to locate and open the app, and choose the correct day. In addition, in the DMKG-App, the timing of the reminders (8 PM) is chosen to increase the salience of the original motivation [23,24] at a time when the ability of the users to perform the behavior is high (if a headache occurs this day, it will most probably already have occurred; most participants will be back from work but not yet too tired). This effect could be further improved by personalizing the time of the reminder based on the time of previous entries, as personalization is a well-established persuasive design principle [25].

Motivation may also decrease with reminder failure, as it becomes more difficult to reach the desired goal (having a good headache documentation) [22,23]. Reminders offer additional possibilities to increase motivation (not implemented in the DMKG-App), for example, making the response to the daily reminder enjoyable by including elements of gamification (like receiving points or seeing interesting animations). Regarding self-determination theory [14], DMKG-App reminders use language emphasizing user autonomy (“Your DMKG-App is ready for today’s entry” instead of “You need to make your entry now” or “Your entry for today is still missing”). The feeling of autonomy could be further increased by allowing the user to choose the timing of the reminders. Up to now, the DMKG-App does not build on social relatedness, which might be an additional possibility to increase the effect of reminders according to self-determination theory [14]. Relatedness could be promoted by including the user’s name in the reminders, by including information like “xxx DMKG-App users have already made the entry for today,” or by using chatbot elements starting a reminder with “Can I help you to make your entry for today,” possibly with personalized reactions like “I am sorry that you had a strong headache today.” However, this carries a risk of bias, so that the effect of such an intervention on data quality would need careful evaluation.

While the reduction of daily entries and the excess attrition of users during the failure period may seem modest, the delay to perform the daily entry increased from 1.90 days in the prefailure period to 2.78 days in the failure period. This is a large effect that will likely increase recall bias. The exact clinical significance of a +0.9 day shift in entry delay is difficult to estimate, as most recall bias research in headache compared headache diaries to retrospective ratings after longer periods such as 1‐3 months [2,26,27]. Results from time-diary research suggest that a 24 hours delay can result in an error of approximately 10% [28]. Our results therefore may have implications for both clinical headache care and scientific use of these data as both rely on data quality. Our data suggest that persons with low headache frequencies and those with high previous adherence are most sensitive to the reminder failure. The first relation seems reasonable as the headache itself might serve as a trigger to make the entry (within theory by Fogg [12]) and motivation (within Fogg or COM-B frameworks [11,12]) may be higher with more frequent headaches as headache-related disability is larger. Indeed, there is some previous evidence that adherence to a headache diary is better on headache days than on headache-free days [29].

On the other hand, in participants with low adherence despite receiving daily reminders, reminders seem to be largely ineffective anyway, possibly explaining why participants with higher adherence in the presence of reminders had a larger drop in adherence when reminders failed. However, there is no literature to support this speculation. In our data, there was also a small effect of males having a larger increase in recording delay with reminder failure than females. There is evidence that women are more socialized to engage in preventive health care and more readily integrate health behavior in their daily routines [30], most likely pointing toward an increased motivation for the behavior. Some (but not all) previous studies on mobile health apps showed better adherence in females than in males [31], and such a relation has also been reported in a headache diary study [32], but the specific effect of reminders has not been investigated. In addition, there is some evidence that males may respond more to gamification elements than women in the health context (eg, [33]). Reminders may be regarded as a basal level of gamification. Further studies are needed to assess gender differences in adherence and personalized interventions may result from this.

One result from our study needs further comment. The attrition rates were generally lower in the previous year (2.8%‐2.9% in the precontrol and postcontrol periods) than in the year where the failure occurred (3.9%‐4.0% in the prefailure and postfailure periods). This is likely due to the fact that the DMKG-App was made freely available in Germany between these 2 time points. Before that, its use was limited to users being treated at a center participating in the DMKG Headache Registry, likely leading to lower attrition rates.

### Comparison to Prior Work

There are not many recent studies investigating adherence to an electronic headache diary, and it is difficult to compare results because of different approaches. An analysis of 1561 users of the Curelator / N1 headache app showed that adherence strongly depended on the setting, being highest for a paid version, intermediate after physician recommendation using a download coupon, and lowest for a free version. In the Curelator / N1 headache analysis, 90-day adherence (proportion of entered days) was 66%, 44% and 34% in the 3 settings, respectively [34]. These values cannot be directly compared to our results showing 70%‐75% adherence, as we included only “active users” in the analysis (see “Methods” section). On the other hand, in a study including 1009 patients of the Leiden Headache Center using the headache center’s e-diary for a minimum of 2 weeks and a median of 181 days, adherence was very high with only 10.8% of the daily entries missing [32]. This is likely due to this study being based on patients from 1 tertiary headache center who were explicitly asked to use the e-diary for their clinical follow-up or study participation. A study with adolescents treated at a tertiary headache center found that over 90 days, 68.7% of the entries within an internet-based headache diary were made on the same day. This rate was higher for headache days with acute medication intake [29]. A study using a headache diary app also providing progressive muscle relaxation that recruited participants at a tertiary headache center found an average of 51 days entered during the first 90 days (adherence 57%), but adherence sharply declined over time [35].

It has been emphasized that there are many factors influencing health-related electronic diary use, including app design (usability, content, feedback on entered data, technical reliability) and user factors (age, gender, digital skills, and symptom load) [4]. Regarding the effect of reminders on diary adherence, there are some reports suggesting that reminders increase use of health-related diaries, but there is a lack of formal investigation and no specific data on headache diaries [4]. Early studies on pain (not headache) diaries showed that reminders increased compliance with a paper diary (ie, entry within ±15 min of assigned time, 3 times daily) from 11% to 29% and use of an electronic diary with reminders further increased compliance to 94% over a 21-day study period [3,36].

Daily entries in headache diaries are crucial for obtaining accurate and reliable data, in both clinical practice and research. Consistent daily tracking enables the identification of patterns, triggers, and treatment effects, allowing for more personalized treatment strategies [2]. Regular use of an electronic headache diary was associated with significant improvements in headache and migraine frequency, intensity, and use of acute medication over a 6-month period [37]. These findings underscore the potential of electronic headache diaries to not only enhance data quality but also to actively support standard headache care by raising awareness for the individual headache disorder. The use of reminders likely can further promote adherence to daily entries and more effective treatment adjustments [37].

### Strengths and Limitations

Strengths of the present study are the large sample size and the comparison with the same periods in the previous year. There also are some limitations. First, the failure period was relatively short, so it remains open if adherence would have deteriorated further or leveled out during a longer period. Because reminders arrived regularly before the failure period, users may have been relying on reminders for their daily entry, and it cannot be excluded that they would have started regular use of the app without reminders after a while. Indeed, the delay to perform the daily entry was mitigated somewhat after the first few days (Figure 2); however, a return to previous values only occurred after the end of the reminder failure. These questions could only be answered by a dedicated study, for example, comparing different reminder failure periods.

Second, total adherence rates determined within the present study may not be generalizable as we included only active users in the analysis. This was necessary to perform a meaningful analysis of the effect of the reminder failure. However, we have performed an additional analysis of the effect of previous adherence on the reaction to the reminder failure, showing that persons with high previous adherence (during a period with reminders) were more sensitive to reminder failure than persons with lower previous adherence. In future, dedicated studies might investigate the effect of reminders on new users specifically within the first weeks of app use–one could speculate that these initial reminders could help users engage in regular entries. It would also be interesting if sending reminders to users after a period of inactivity can bring them back to regular use.

Third, although the present study showed that daily reminders increase adherence to an electronic headache diary, it remains an open question what type of reminder would have the largest effect on adherence, a question that has also been discussed in other mobile health apps [38]. Future research could further refine understanding of reminders in headache diaries by comparing groups randomly assigned to different conditions, for example, receiving reminders vs no reminders from the beginning of app use vs reminders ceasing after a fixed time of app use with a longer follow-up period without reminders. They could also compare different types of reminders directed at different effects, for example, increasing ability and/or motivation in addition to serving as a prompt according to behavioral theory by Fogg [12].

### Conclusion

This is the first study on the effect of daily reminders in a digital headache diary app. Present results suggest that daily reminders are an effective means to improve adherence to electronic headache diaries, specifically by increasing the number of documented days and by decreasing the delay to make the daily entry. Males, persons with low headache frequencies, and high previous adherence were more sensitive to reminder failure. These results contribute to our knowledge on compliance with digital health apps and point out means to improve adherence and groups especially benefiting from reminders.

## Supplementary material

10.2196/73259 Multimedia Appendix 1 Supplementary tables and interactions with age, gender, headache frequency, and previous adherence rates.

## Abbreviations

DMKG: Deutsche Migräne- und Kopfschmerzgesellschaft e.V. (German Migraine and Headache Society)
DMKG-App: the headache diary app of the German Migraine and Headache Society
DRKS: Deutsches Register Klinischer Studien (German Clinical Trials Register)
EU: European Union
GDPR: General Data Protection Regulation
