# Supplementary material for: Daily Reminders Increase Adherence to an Electronic Headache Diary (DMKG-App): Retrospective Longitudinal Cohort Study
Source: JMIR Mhealth Uhealth. 2026 Sep 14;14:e73259. doi: 10.2196/73259 (PMC13574331; doi:10.2196/73259)
Supplement: Supplementary file 1 [file mhealth-v14-e73259-s001.docx]

**Supplementary Table 1:** Interaction with age and gender

| **Age** |  | **Interaction age x period** | **<37 years (n = 1882)** | **≥37 years (n = 2354)** |
| --- | --- | --- | --- | --- |
|  |  |  | Difference  from pre-failure to failure period  (95% CI) | |
|  | Number of recorded days (per 14-day period) | F = .2, df = 2, *P*=.813 | -1.21 (0.92-1.59) | -1.08 (0.82-1.32) |
|  | Delay (days) | F = .2, df = 2, *P*=.784 | +0.88 (0.87-0.90) | +0.88 (0.87, 0.89) |
|  | Attrition rate | z = -1.37, *P*=.170 | +1.0% (-0.6-2.4) | +1.6% (0.5-2.7) |
|  | | | | |
| **Gender** |  | **Interaction gender x period** | **Male (n = 760)** | **Female (n = 3460)** |
|  |  |  | Difference  from pre-failure to failure period  (95% CI) | |
|  | Number of recorded days (per 14-day period) | F = 1.7, df = 2, *P*=.184 | -1.25 (0.78-0.72) | -1.11 (0.90-1.32) |
|  | **Delay (days)** | **F = 5.0, df = 2, *P*=.009** | **+1.03 (1.00-1.06)** | **+0.85 (1.84-1.86)** |
|  | Attrition rate | z = 0.6, *P*=.606 | +2.3% (-0.1-4.6) | +1.1% (0.1-2.1) |

This was a longitudinal analysis of data from the DMKG App, a headache diary app freely available in Germany, comparing a period where no daily reminders were sent with periods with reminders. Three outcome parameters were considered: number of days recorded in the headache diary, delay to make the daily entry, and attrition rate. Interactions between age and period and gender an period are shown, respectively. Age groups were: younger <37 years, older ≥37 years; gender groups were: male and female; periods were: pre-failure, failure, post-failure. Significant results are marked in bold.

**Supplementary Table 2:** Interaction with headache frequency and with previous adherence rate

| **Headache frequency** |  | **Interaction headache frequency x period** | **Low frequency**  **(0-7 days/month)**  **(n = 941)** | **Medium frequency**  **(8-14 days/month)**  **(n = 668)** | **High frequency**  **(≥15 days/month)**  **(n = 378)** | **Statistics (low vs. medium)** | **Statistics (low vs. high)** | **Statistics (medium vs. high)** |
| --- | --- | --- | --- | --- | --- | --- | --- | --- |
|  |  |  | Difference from pre-failure to failure period (95% CI) | | |  |  |  |
|  | **Number of recorded days (per 14-day period)** | **F = 3.3, df = 4,**  ***P*=.011** | -1.43  (-1.76 to -1.10) | -0.79  (-1.16 to -0.42) | -0.88  (-1.37 to -0.39) | **F = 5.5, df=2,**  ***P*=.004** | **F = 3.1, df = 2,**  ***P*=.044** | F <0.1, df = 2,  *P*=.956 |
|  | **Delay (days)** | **F = 18.9, df = 4,**  ***P*<.001** | +1.17  (+1.15 to +1.19) | +0.87  (+0.85 to +0.89) | +0.60  (+0.58 to +0.62) | **F = 10.8, df=2,**  ***P*<.001** | **F = 36.0, df = 2,**  ***P*<.001** | **F = 8.5, df = 2,**  ***P*<.001** |
|  | Attrition rate | z = -0.64, *P*=.525 | +2.6%  (+0.10 to +4.23) | +1.1%  (-0.52 to +2.65) | +1.5%  (-0.71 to +3.63) | - | - | - |
|  | | | | | | | | |
| **Previous adherence rate** |  | **Interaction previous adherence x period** | **Low previous adherence**  **(0-14 of 31 days)**  **(n = 489)** | **Medium previous adherence (15-30 of 31 days)**  **(n = 503)** | **High previous adherence**  **(31 of 31 days)**  **(n = 1650)** | **Statistics (low vs. medium)** | **Statistics (low vs. high)** | **Statistics (medium vs. high)** |
|  |  |  | Difference from pre-failure to failure period (95% CI) | | |  |  |  |
|  | **Number of recorded days (per 14-day period)** | **F = 5.8, df = 4,**  ***P*<.001** | +0.13  (-0.60 to 0.86) | -0.68  (-1.22 to -0.14) | -1.21  (-1.44 to ‑0.98) | F = 1.8, df = 2,  *P*=.171 | **F = 11.5, df=2,**  ***P=*.002** | F <2.4, df = 2,  *P*=.088 |
|  | **Delay (days)** | **F = 10.7, df = 4,**  ***P*<.001** | +0.77  (+0.73 to +0.81) | +0.81  (+0.78 to +0.84) | +0.98  (+0.97 to +0.99) | **F = 6.7, df=2,**  ***P*=.002** | **F = 23.4, df = 2,**  ***P*<.001** | **F =3.7, df = 2,**  ***P*=.029** |
|  | **Attrition rate** | **z = -4.22, *P*<.001** | -1.9%  (-4.4 to +0.5) | -0.6%  (-2.8 to +1.7) | +2.7%  (+1.6 to +3.8) | z = 0.70, *P*=.483 | **z = 4.05, *P*<.001** | **z = 3.20, *P*=.001** |

Significant results are marked in bold.
